# Urinary TIMP-2 and IGFBP7 as Early Biomarkers of Acute Kidney Injury and Renal Recovery following Cardiac Surgery

**DOI:** 10.1371/journal.pone.0093460

**Published:** 2014-03-27

**Authors:** Melanie Meersch, Christoph Schmidt, Hugo Van Aken, Sven Martens, Jan Rossaint, Kai Singbartl, Dennis Görlich, John A. Kellum, Alexander Zarbock

**Affiliations:** 1 Department of Anesthesiology, Intensive Care and Pain Medicine, University of Münster, Münster, Germany; 2 Department of Cardiac Surgery, University of Münster, Germany; 3 Department of Anesthesiology, Penn State College of Medicine, Hershey, Pennsylvania, United States of America; 4 Institute of Biostatistics and Clinical Research, University of Münster, Münster, Germany; 5 Center for Critical Care Nephrology, CRISMA Center, Department of Critical Care Medicine, University of Pittsburgh, Pennsylvania, United States of America; University of Tübingen, Germany

## Abstract

**Background:**

Difficulties in prediction and early identification of (acute kidney injury) AKI have hindered the ability to develop preventive and therapeutic measures for this syndrome. We tested the hypothesis that a urine test measuring insulin-like growth factor-binding protein 7 (IGFBP7) and tissue inhibitor of metalloproteinases-2 (TIMP-2), both inducers of G1 cell cycle arrest, a key mechanism implicated in acute kidney injury (AKI), could predict AKI in cardiac surgery patients.

**Methods:**

We studied 50 patients at high risk for AKI undergoing cardiac surgery with cardiopulmonary bypass (CPB). Serial urine samples were analyzed for [TIMP-2]*[IGFBP7] concentrations. The primary outcome measure was AKI as defined by international consensus criteria following surgery. Furthermore, we investigated whether urine [TIMP-2]*[IGFBP7] could predict renal recovery from AKI prior to hospital discharge.

**Results:**

26 patients (52%) developed AKI. Diagnosis based on serum creatinine and/or oliguria did not occur until 1–3 days after CPB. In contrast, urine concentration of [TIMP-2]*[IGFBP7] rose from a mean of 0.49 (SE 0.24) at baseline to 1.51 (SE 0.57) 4 h after CPB in patients who developed AKI. The maximum urinary [TIMP-2]*[IGFBP7] concentration achieved in the first 24 hours following surgery (composite time point) demonstrated an area under the receiver-operating characteristic curve of 0.84. Sensitivity was 0.92, and specificity was 0.81 for a cutoff value of 0.50. The decline in urinary [TIMP-2]*[IGFBP7] values was the strongest predictor for renal recovery.

**Conclusions:**

Urinary [TIMP-2]*[IGFBP7] serves as a sensitive and specific biomarker to predict AKI early after cardiac surgery and to predict renal recovery.

**Clinical Trial Registration Information:**

www.germanctr.de/, DRKS-ID: DRKS00005062

## Introduction

Acute kidney injury (AKI) is a common and serious complication after cardiac surgery [1]. It may occur in over 40% of adults, with 1–5% requiring renal replacement therapy (RRT) [2]–[4]. After cardiac surgery, small creatinine increases of 20–25% from preoperative baseline are associated with adverse outcomes [3], [5]. The mortality in cardiac surgery patients with a severe, RRT-requiring AKI can be as high as 60% [3], [4], [6]. Although some clinical tools and scores exist to predict and stratify AKI, we are still lacking biomarkers to predict AKI and recovery from AKI early enough for interventions to be likely effective. The incidence and severity of AKI and patients outcome have not changed in recent years [1], [7], [8].

Currently, diagnosing and staging of AKI are exclusively based on elevations in serum creatinine and/or decreases in urine output. Serum creatinine, however, is widely known to be insensitive to acute changes in kidney function [9]. Serum creatinine concentrations neither accurately reflect the glomerular filtration rate nor do they point to the degree of tubular injury [6], [10]. Therefore, serum creatinine values are poorly qualified to detect AKI in the early period after cardiac surgery [11]. The same is true for postoperative oliguria, which can be influenced by a myriad of factors including volume status and use of diuretics. At the very least, several hours are needed to define oliguria. Several attempts to treat AKI have failed, perhaps in part because therapies were initiated too late in the presence of an already established acute tubular necrosis (ATN) [12].

Therefore, identifying biomarkers to predict the development and severity of AKI early after cardiac surgery has been an important goal for over a decade. Several biomarkers including interleukin (IL)-18 [13], neutrophil gelatinase-associated lipocalin (NGAL) [14], cystatin c [15], and kidney injury molecule-1 (KIM-1) [16] have been studied. However, the area under the curve (AUC) and therefore the suitability of these biomarkers to predict AKI after cardiac surgery were fairly low (0.65 for KIM-1 [17], 0.67 for NGAL [17], and 0.71 for cystatin c [15]).

AKI affects different complex cellular and molecular pathways involving inflammatory, interstitial, endothelial, and epithelial cells. These mechanisms comprise immunity, inflammation, apoptosis, and cell cycle pathways. A recent study showed that renal tubular cells enter a period of G1 cell-cycle arrest after inducing ischemia [18] or sepsis [19]. IGFBP7 and TIMP-2 are both involved in G_1_ cell cycle arrest during the early phase of cell injury [20]–[22]. The G_1_ cell cycle arrest may prevent the division of cells with damaged DNA until the DNA damage is repaired [21]. In the Sapphire study [23], it was demonstrated that the AUC values to predict the development of AKI (AKIN stage 2 or 3) in critically ill patients within 12 hours were 0.76 for IGFBP7 and 0.79 for TIMP-2. Multiplication of the two markers ([TIMP-2]*[IGFBP7]) resulted in an even higher AUC (0.80) and was significantly superior to all previously described markers of AKI. Moreover, [TIMP-2]*[IGFBP7] significantly improved risk prediction when added to clinical scoring systems.

Therefore the aim of the current study was to test the hypothesis that urinary [TIMP-2]*[IGFBP7] can predict AKI early after cardiac surgery and that urinary [TIMP-2]*[IGFBP7] can function as a prognostic marker in patients with established AKI providing information about the likelihood of recovery.

## Materials and Methods

### Patients and methods

**Figure 1:**
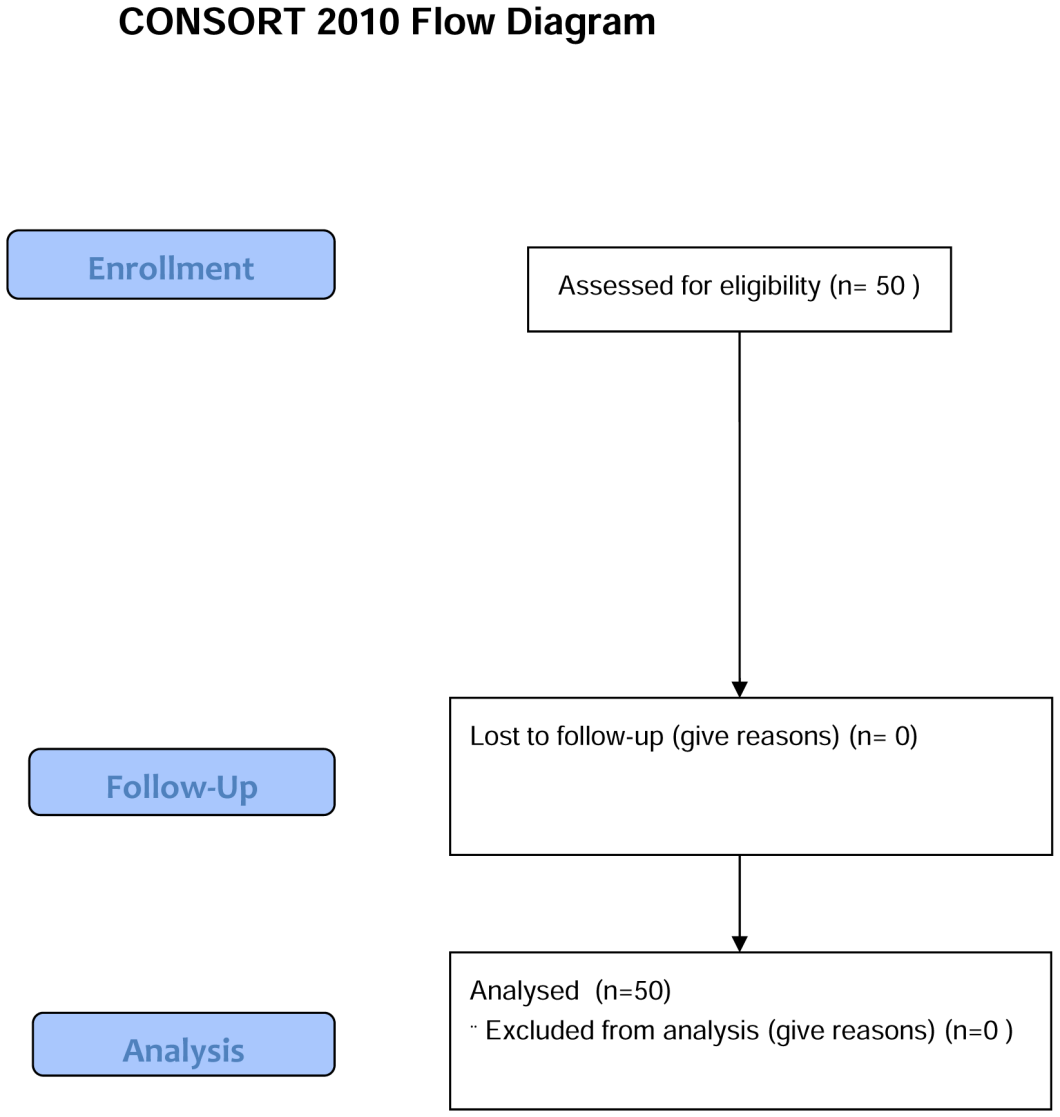
CONSORT 2010 Flow Diagram.

The study was approved by the institutional review board of the University of Münster. We used the Standards for Reporting of Diagnostic Accuracy (STARD) statement for planning and conducting the study and preparing the manuscript [24]. We screened all patients admitted to the University of Münster Cardiac Surgery service for cardiac surgery with CPB between June 2013 and September 2013 (Figure 1: CONSORT 2010 Flow Diagram). Patients with a Cleveland Clinic Foundation Score [8] of 6 or more were eligible for enrollment. All patients eligible for enrollment were approached. Exclusion criteria included (1) pregnancy; (2) post-renal transplantation; (3) immunosuppressive therapy; and (4) patients receiving corticosteroid therapy with a change in their dose within the past 2 weeks. Written informed consent was obtained from all patients at the time of enrollment. All patients were prospectively followed from enrollment. Urine samples were collected at predetermined time points: on the day of the surgery preoperatively (immediately post-anesthesia induction), 4 h, 12 h, and 24 h after coming off CPB. At the same time points, creatinine concentrations in the urine were measured. Serum creatinine levels were measured preoperatively, 4 h, 12 h, 24 h, 48 h, 72 h, and at the time of hospital discharge. All patients received routine standard of care during the study period. ICU and hospital discharge were conducted at the discretion of the treating clinicians, not involved in the study and not aware of study data. Cardiac function and volume status were ascertained by clinical judgment combined with transthoracic echocardiography (TTE). A TTE examination was performed routinely on all patients on the day of surgery (2 to 4 h after admission to the ICU) and on day 1 following surgery. Patients were considered to have completed the study at the time of hospital discharge. Urine samples were immediately centrifuged, urine supernatants were frozen, stored at ≤−70°C and thawed immediately prior to analysis.

Preoperative patient characteristics, significant intraoperative risk factors and peri- and postoperative complications were recorded. All clinical decisions were made by the responsible cardiac surgeon, cardiac anesthetist, and/or intensivist.

### Study endpoints

AKI status was classified using the RIFLE [25] or AKIN criteria [26] together as described in the recent KDIGO international guideline [27] based on the serum creatinine (sCR) and urine output (UO) available in the hospital record. The primary end point was the development of AKI after cardiac surgery. The reference value for serum creatinine was obtained as follows: if at least five values were available the median of all values available from six months to one day prior cardiac surgery was used. Otherwise, the creatinine value determined one day before cardiac surgery was used. Serum creatinine was measured daily during the entire hospital stay. Urine output was recorded hourly during the ICU stay and twice daily until hospital discharge. The secondary end point was renal recovery from established AKI defined as a serum creatinine value at hospital discharge equal to or lower than the preoperative creatinine value. As per hospital standard of care, all patients were discharged to a rehabilitation facility, meeting predefined discharge criteria. Other end points included length of ICU and hospital stays. Furthermore, we recorded variables known to impact AKI risk: age, sex, CPB time, cross-clamp time, hypertension, congestive heart failure, diabetes, peripheral vascular disease, previous cardiothoracic surgery, preoperative creatinine clearances estimated by both Cockcroft and Gault and Modification of Diet in Renal Disease equations [28], serial plasma and urine creatinine, and urea nitrogen.

### Biomarkers

TIMP-2 and IGFBP7 were measured with the NephroCheck™ Test (Astute Medical, San Diego, CA, USA). The NephroCheck Test is a point-of-care test which was developed to simultaneously measure urine [TIMP-2]*[IGFBP7], whereas [TIMP-2]*[IGFBB7] indicates the multiplication of both biomarkers. To measure both biomarker concentrations in the urine, 100 µl urine is required and it takes 20 minutes at the bedside to get the result. Urine NGAL, a previously described biomarker of AKI, was measured with a commercially available assay (Dianova, Hamburg, Germany) according to the manufacturer's protocol.

### Statistical analysis

For the primary analysis, that is the difference between the urine [TIMP-2]*[IGFBP7] levels in patients with AKI or without AKI, we applied a Mann-Whitney-U-test. Based on the published results on [TIMP-2]*[IGFBP7] [23], we aimed to detect a difference in 1 unit in [TIMP-2]*[IGFBP7] with a power of 90%. Assuming an effect size of 1, a sample size of 26 patients per group is necessary. Power calculation was performed with nQuery Advisor (Version 7).

The study population was described by absolute and relative frequencies, mean and standard error, where appropriate. To test for a difference in age between AKI and non-AKI patients a t-Test was applied. All other continuous variables were tested using Mann-Whitney-U-tests due to lacking normality. The association between categorical variables and the two groups (AKI vs. non-AKI) was tested with Chi-Squared-Tests and, in case of previous heart surgery and chronic obstructive pulmonary disease, with Fisher's Exact Test.

The analysis of [TIMP2]*[IGFBP7] time course data was performed independently for each time point using Mann-Whitney-U-Tests. In each group the difference between the respective time point and the baseline, i.e., pre-CPB, was tested, as well as the location difference between the group at each individual time point.

For all tests p-values ≤0.05 were considered significant. No adjustment for multiple testing was performed. Results were considered exploratory. The presented findings may be used to generate new hypotheses.

To analyze the predictive power of selected biomarkers receiver operating characteristic curves (ROC) were calculated and the area under the ROC curve (AUC) was determined. 95% confidence intervals (CI) were reported. For selected thresholds of [TIMP2]*[IGFBP7] sensitivities, specificities, positive predictive values (PPV) and negative predictive values (NPV) were reported for each time point and the first 24 h post-operatively combined (composite time point). Descriptive statistics, statistical tests, and ROC analyses were performed using SPSS 21 (IBM SPSS Statistics for Windows, Version 21.0. Armonk, NY: IBM Corp.).

To evaluate the additional information [TIMP-2]*[IGFBP7] gives for risk classification in comparison to a pure clinical model we calculated the integrated discrimination improvement (IDI) and the category-free net reclassification index (cfNRI) [29]. IDI and cfNRI have been calculated using the package PredictABEL [30] in R (R version 3.0.0, April 2013. R Foundation for Statistical Computing, Vienna, Austria). P-values and 95%-confidence intervals (CI) for IDI and cfNRI are provided by the function reclassification and are determined using a z-transformation of the statistics.

## Results

### Patient characteristics and study endpoints

We recruited 50 patients and analyzed 50 patients. Thus, 50 patients were included in the study. The clinical characteristics of the 50 subjects are provided in Table 1.

**Table 1:** Clinical characteristics of subjects (n = 50).

	Total (n = 50)	AKI (n = 26)	Non AKI (n = 24)	p-value
Age	71±12	70±12	72±11	0.534
Gender				0.616
Male [%]	33 (66)	18 (69.2)	15 (62.5)	
Female [%]	17 (34)	8 (30.8)	9 (37.5)	
Preoperative creatinine [mg/dl]	1.33±0.3	1.37±0.4	1.28±0.21	0.695
eGFR [ml/min per 1.73 m^2^]	51±11	50±14	53.8±12	0.494
Comorbidities				
Hypertension [%]	48 (96)	24 (92.3)	24 (100)	0.166
Congestive Heart Failure [%]	46 (92)	24 (92.3)	22 (91.7)	0.933
Diabetes [%]	20 (40)	12 (46.2)	8 (33.3)	0.355
COPD [%]	15 (30)	12 (46.2)	3 (12.5)	0.009
Chronic kidney disease [%]	15 (30)	11 (42.3)	4 (16.7)	0.048
Previous heart surgery [%]	6 (12)	6 (23.1)	0 (0)	0.023
Left ventricular EF<35% [%]	12 (22)	8 (30.8)	3 (12.5)	0.119
CPB time [minutes]	140±60	149±74	129±38	0.818
X-clamp [minutes]	98±50	110±63	85±26	0.358
APACHE on day one	10±5	12±5	8±3	0.001
Length of ICU stay [days]	8±2	12±3	4±1	0.001
Length of hospital stay [days]	19±2	24±3	14±1	0.001

Data are expressed as mean ± s.e. or number (percentage).

eGFR, estimated glomerular filtration rate; COPD, chronic obstructive pulmonary disease; EF, ejection fraction; CPB, cardiopulmonary bypass; APACHE, Acute Physiology And Chronic Health Evaluation; ICU, intensive care unit; p≤0.05 significant.

Of the 50 study subjects, 26 (52%) developed postoperative AKI (the primary study end point) as defined by the KDIGO criteria. In nine patients, AKI was diagnosed first by increases in serum creatinine while in the remaining 17 patients, urine output criteria were met first (Table 2). In all patients, hypovolemia was excluded by clinical assessment using echocardiography. The mean preoperative serum creatinine for all patients was 1.32 mg/100 ml (median = 1.20 mg/100 ml), consistent with a mean preoperative Modification of Diet in Renal Disease-estimated glomerular filtration rate (eGFR) of 50.4 ml/min per 1.73 m^2^ body surface area. Baseline serum creatinine (mg/100 ml; mean (standard error)) was 1.28±0.21 in the group that did not develop AKI (n = 24), not significantly different from the AKI group (1.36±0.40, n = 26, p = 0.342). There was also no significant difference in baseline eGFR values between the two groups (p = 0.286; Table 1). The peak serum creatinine (mg/100 ml) postoperatively was significantly higher in the AKI group than in the group that did not develop AKI (1.38±0.54 vs 1.06±0.27, respectively; p = 0.015).

**Table 2:** Number and reasons of AKI.

	Number	Reason for AKI	
		Increased creatinine	Oliguria
AKIN stage 1	19	9	10
AKIN stage 2	6	0	6
AKIN stage 3	1	0	1

There were significantly longer intensive care unit (ICU, p = 0.001) and hospital stays (p = 0.001) in the AKI group (Table 1). Finally, the AKI group had a higher APACHE score on day 1 (p = 0.001) than those without AKI (Table 1).

### Biochemical value of urinary [TIMP-2]*[IGFBP7] for early diagnosis of AKI

**Figure 2:**
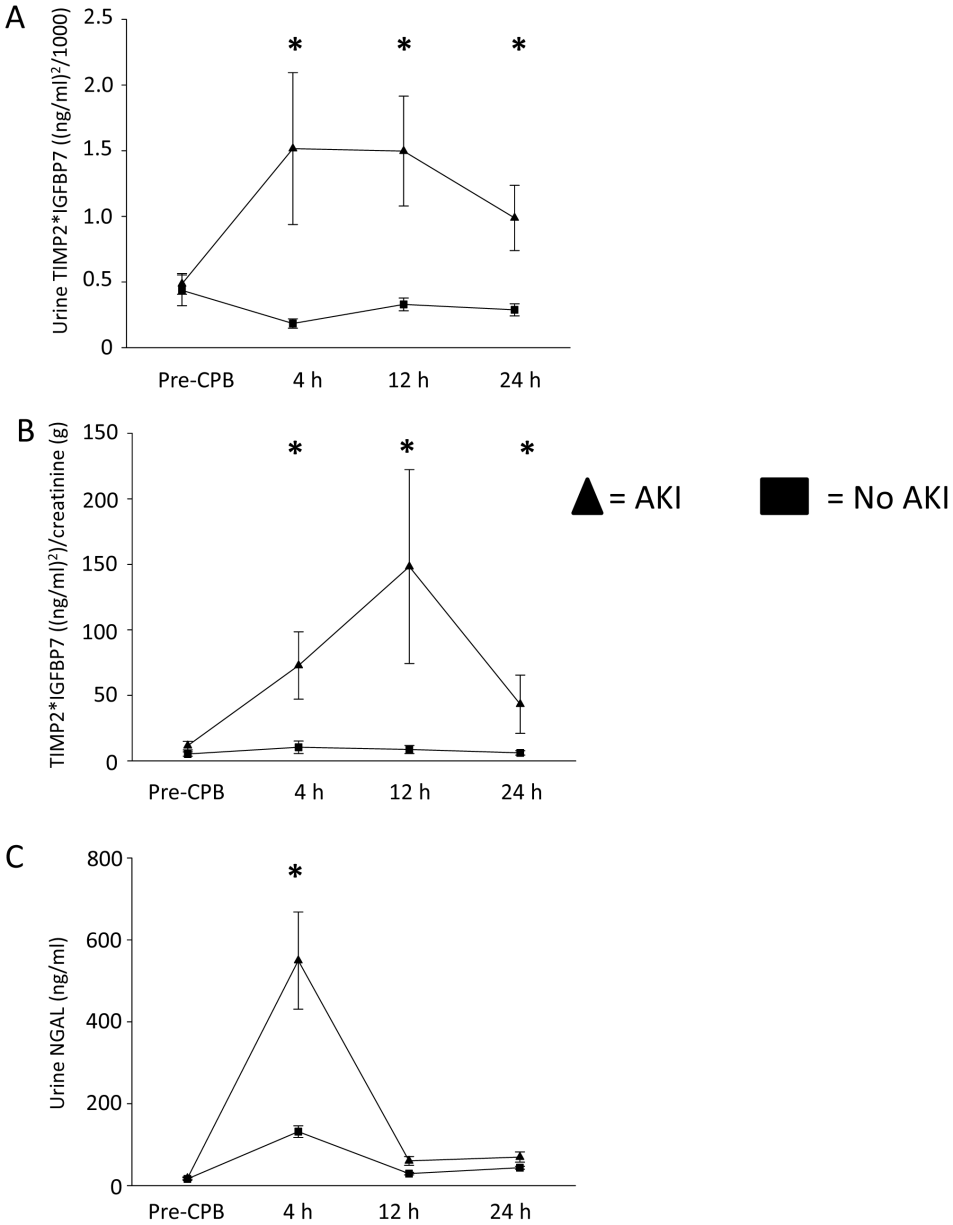
Analysis of urine [TIMP-2]*[IGFBP7]. (A) Graph shows mean urine [TIMP-2]*[IGFBP7] concentrations at various time points before and after cardiopulmonary bypass. (B) Graph shows urine [TIMP-2]*[IGFBP7] corrected for urine creatinine excretion. (C) Graph shows mean urine NGAL concentrations at various time points before and after cardiopulmonary bypass. Error bars are SE. Asterisks (*) denote significant differences (p≤0.05) between groups (AKI, non-AKI) at the respective time point.

In the 24 patients who never developed AKI, no significant increase was noted in urinary [TIMP-2]*[IGFBP7] concentrations at any time point after cardiopulmonary bypass compared to the preoperative measurement (pre-CPB) (all p-values >0.05). By contrast, those who subsequently developed AKI had a striking rise in urinary [TIMP-2]*[IGFBP7] concentrations at all time points compared to pre-CPB (all p-values ≤0.05; Figure 2A). The pattern of urinary [TIMP-2]*[IGFBP7] excretion was characterized by a peak very early after the precipitating event followed by a lesser but sustained increase over the entire duration of the study (Figure 2A). These results remained unchanged when urinary [TIMP-2]*[IGFBP7] concentration was normalized for urinary creatinine excretion although the time-profile of the point estimates for the [TIMP-2]*[IGFBP7] values shifted slightly (Figure 2B). In the 26 patients who developed AKI, urinary NGAL significantly increased at 4 h after CPB (p = 0.001) followed by a rapid decrease back to baseline at 12 h after CPB (Figure 2C).

### Performance of urinary [TIMP-2]*[IGFBP7] for diagnosis of AKI

**Figure 3:**
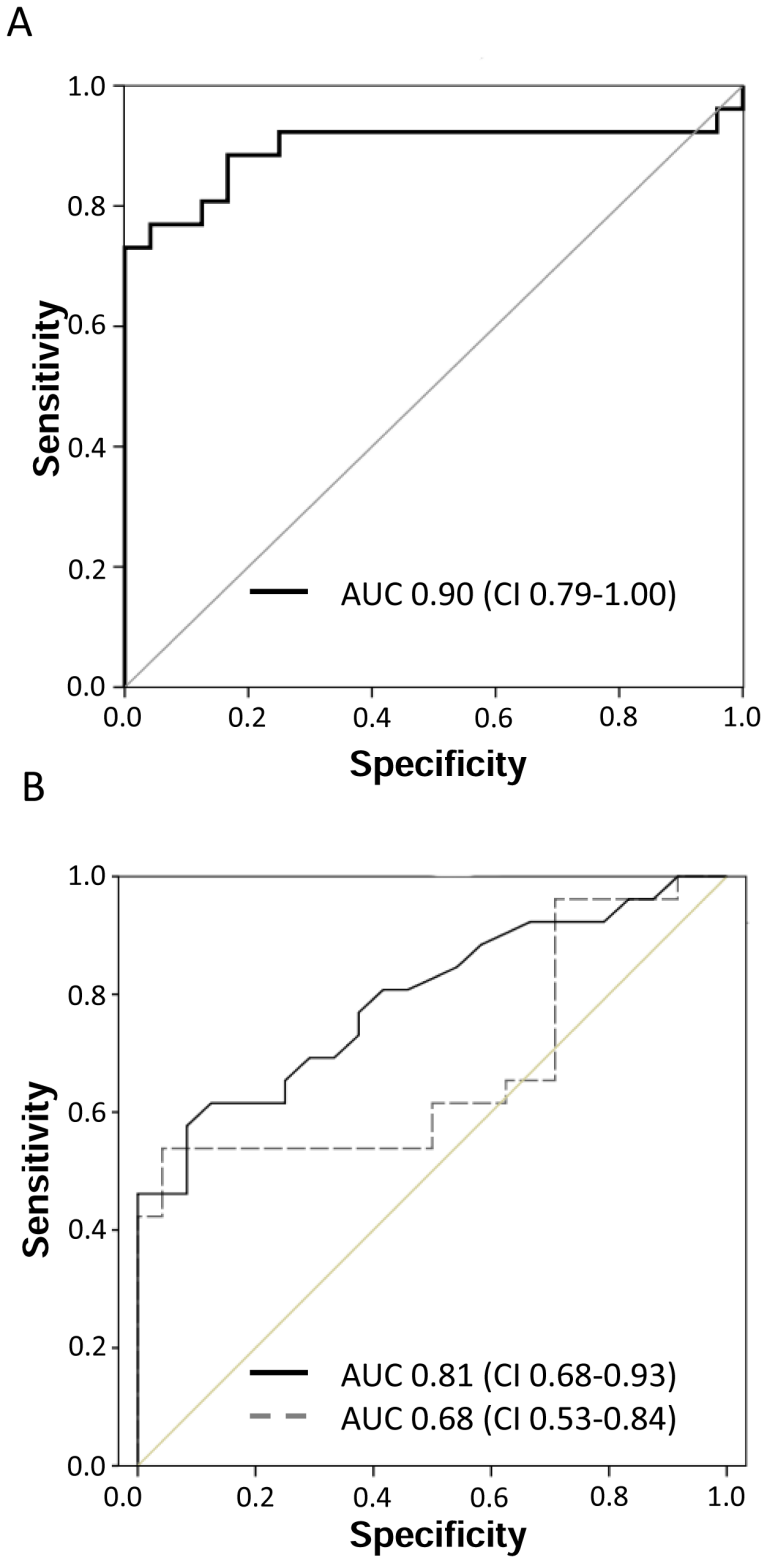
ROC curves for the maximum early composite and the 4 (A) This figure displays the receiver operating characteristic (ROC) curve for the maximum early composite (maximum value from the first 24 postoperative hours) for [TIMP-2]*[IGFBP7]. (B) This figure displays the receiver operating characteristic (ROC) curves for the 4 h values of [TIMP-2]*[IGFBP7] (black solid line) and NGAL (gray dashed line).

A composite time point consisting of the maximum urinary [TIMP-2]*[IGFBP7] concentration attained during the first 24 h after CPB outperformed all individual time points (AUC: 0.90, CI: 0.79–1.00; Figure 3A). For urine [TIMP-2]*[IGFBP7], the area under the ROC curve was 0.81 (CI: 0.68–0.93) at 4 h after CPB (Figure 3B), whereas the area under the ROC curve for urine NGAL was 0.68 (CI: 0.53–0.84). Table 3 lists the derived sensitivities, specificities, and predictive values at different cutoff concentrations. For urine [TIMP-2]*[IGFBP7], a cutoff of 0.3 yielded good sensitivity and specificity at 4 h after CPB.

**Table 3:** [TIMP-2]*[IGFBP7] test characteristics at different cutoff values.

	Sensitivity	Specificity	PPV	NPV
Composite time*				
0.3	0.92	0.66	0.73	0.88
0.4	0.92	0.67	0.75	0.99
0.5	0.92	0.81	0.80	0.90
0.6	0.81	0.83	0.84	0.80
0.7	0.81	0.91	0.91	0.81
4 h				
0.3	0.80	0.83	0.80	0.83
0.4	0.62	0.88	0.84	0.68
0.5	0.54	0.92	0.86	0.65
0.6	0.46	0.92	0.86	0.61
0.7	0.46	1.0	1.0	0.63
12 h				
0.3	0.85	0.50	0.65	0.75
0.4	0.77	0.75	0.77	0.75
0.5	0.65	0.83	0.81	0.69
0.6	0.58	0.92	0.88	0.67
0.7	0.54	0.92	0.88	0.65
24 h				
0.3	0.73	0.58	0.66	0.67
0.4	0.62	0.75	0.73	0.64
0.5	0.58	0.83	0.79	0.65
0.6	0.42	0.88	0.79	0.58
0.7	0.27	0.96	0.88	0.55

* composite time: maximum urinary [TIMP-2]*[IGFBP7] concentration ((ng/ml)^2^/1000) achieved in the first 24 hours following surgery; PPV, positive predictive value; NPV, negative predictive value.

### Prediction of renal recovery from AKI with urinary [TIMP-2]*[IGFBP7]

**Figure 4:**
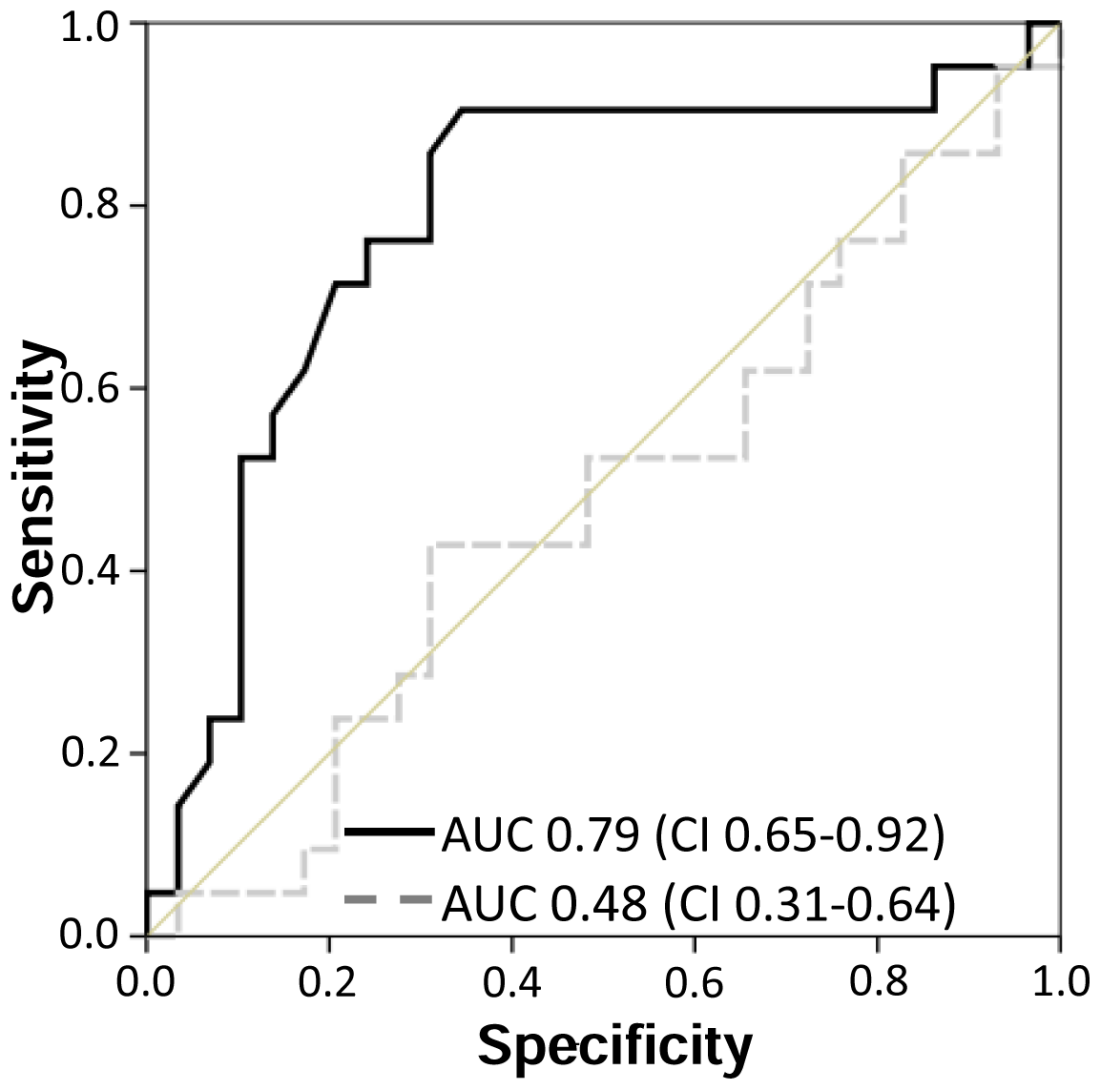
ROC curve for recovery from AKI after cardiac surgery. This figure displays the area under the curve (AUC) for predicting renal recovery. [TIMP-2]*[IGFBP7] (black solid line) and urine neutrophil gelatinase-associated lipocalin (NGAL, gray dashed line).

Next, we assessed the relationship between a decline in urinary biomarker concentrations between 4 and 24 h after surgery and renal recovery, defined as a serum creatinine value at hospital discharge equal to or lower than that at baseline (Figure 4). For [TIMP-2]*[IGFBP7], the area under the ROC curve for the difference between the two values was 0.79 (CI: 0.65–0.92) (Figure 4). The area under the ROC curve for the difference between the two NGAL values was 0.48 (CI: 0.31–0.64). Combination of NGAL with [TIMP-2]*[IGFBP7] did not improve the area under the ROC curve (data not shown).

### Additional information from biomarkers over clinical variables

We also investigated whether [TIMP-2]*[IGFBP7] enhances predictive ability over clinical variables. [TIMP-2]*[IGFBP7] significantly improved risk prediction when added to a six-parameter clinical model for the primary endpoint, using time to event, IDI and cfNRI analyses (Table 4 and 5). All analyses showed significant enhancement by the addition of [TIMP-2]*[IGFBP7] with [TIMP-2]*[IGFBP7] remaining strongly associated with AKI in all models.

**Table 4:** Logistic Regression Risk Models for [TIMP-2]*[IGFBP7] and Clinical Covariates.

	Reference Risk Model		New Risk Model (addition of [TIMP-2]*[IGFBP7] to reference risk model)^4^	
Variable	Odds ratio^3^	p-value	Odds ratio^4^	p-value
**Diabetes mellitus**	1.12 (0.15–8.61)	0.92	3.60 (0.10–128.42)	0.48
**APACHE III Score**	1.74 (1.23–2.69)	0.01	2.62 (1.04–6.57)	0.04
**Ejection fraction**	10.05 (0.67–150.72)	0.09	18.76 (0.16–2143.76)	0.02
**Baseline creatinine**	0.06 (0.01–3.074)	0.16	0.02 (0–120.37)	0.36
**X-Clamp time**	1.01 (0.99–1.04)	0.30	0.99 (0.97–1.03)	0.77
**COPD**	40.28 (2.2–737.82)	0.01	30.49 (0.81–1146.64)	0.07
[TIMP-2]*[IGFBP7] 1 **^,^** 2	Not included in model		1.72 (1.02–2.91)	0.04

1 [TIMP-2]*[IGFBP7] was log10 transformed.

2 Odds ratio for [TIMP-2]*[IGFBP7] is modeled for change of 0.1 unit in log10 units.

3 95%-confidence interval given in brackets.

4 Adding [TIMP-2]*[IGFBP7] improves the model significantly (p = 0.001, likelihood ratio test).

**Table 5:** Integrated discrimination improvement (IDI) and category-free net reclassification improvement (cfNRI) for [TIMP-2]*[IGFBP7].

Statistic	Value	p-value	95%-CI
**IDI**	0.207	0.0015	(0.079–0.336)
**cfNRI**	1.513	<0.001	(1.122–1.905)
**AUC (reference model)**	0.910	<0.001	(0.82–0.99)
**AUC (new model)**	0.967	<0.001	(0.92–1.00)
**AUC (difference)**	0.907	<0.001	(0.80–1.00)

IDI and cfNRI refer to the improved classification of patient using the reference model and the new model (reference model+[TIMP-2]*[IGFBP7]). AUC refers to the area under the receiver operator characteristic curve calculated from the model prediction against the outcome (AKI vs. no AKI). AUC difference refers to the ROC analysis using the difference between predicted probabilities for each patient. AUCs have been calculated in SPSS. IDI and cfNRI have been calculated in R using package PredictABEL. P-values and 95% confidence intervals (CI) for IDI and cfNRI are provided by the function reclassification and are determined by a z-score transformation.

## Discussion

AKI remains a common and serious clinical problem that greatly adds to morbidity and mortality in both surgical and medical patients. The KDIGO clinical practice guideline for AKI [27] recommends risk assessment for all critically ill patients, acknowledging that risk assessment is difficult. Clinical measures, such as increased serum creatinine or decreased urine output, are not suitable for risk assessment because these changes often occur late after organ injury. Even in the relatively homogenous setting of cardiac surgery, there is a paucity of biomarkers to predict AKI. Therefore, there is a great interest to identify biomarkers which can accurately and reliably predict AKI as well as recovery from AKI early after cardiac surgery.

To test such biomarkers, we focused on patients at high risk for AKI. To identify these patients we used the Cleveland Clinic Foundation Score [8]. This score includes different risk categories (e.g. gender, emergency surgery, type of surgery) and has been shown to correlate with dialysis-dependent AKI following cardiac surgery [8]. An important feature of this study is that we also included patients with preexisting chronic kidney disease (CKD), because it has been shown that other biomarkers have limited informative value in patients with CKD [31].

Recently, results from a multicenter study of discovery and validation of two novel biomarkers of AKI, TIMP-2 and IGFBP7, in critically ill patients were reported [23]. The biomarkers were measured in the urine of critically ill patients after ICU admission, and the primary end point of that study was moderate to severe AKI (KDIGO stage 2 to 3) within 12 hours of sample collection. Urine [TIMP-2]*[IGFBP7] showed a better performance in predicting AKI compared to all previously described markers [23]. Here, we demonstrate that [TIMP-2]*[IGFBP7] concentration in the urine of patients undergoing cardiac surgery has a high sensitivity and specificity in predicting AKI after cardiac surgery. The AUC of [TIMP-2]*[IGFBP7] was higher compared to the AUC of NGAL. However, combining NGAL and [TIMP-2]*[IGFBP7] did not increase the predictability of AKI (data not shown). As there are currently no interventions to treat AKI, it becomes even more important to identify patients at high risk for AKI and to diagnose AKI as early as possible. Thereby, necessary steps can be taken to prevent further worsening of already existing damage, e.g. initiating the KDIGO bundle [27]. In contrast, patients with [TIMP-2]*[IGFBP7] concentrations below 0.4 in the postoperative period developed no AKI and had a briefer stay in the ICU and hospital compared to patients with high concentrations.

The recovery rate of renal function is low in critically ill patients with AKI [32]. This fact emphasizes that incomplete renal recovery is a common problem in patients surviving severe AKI [33]. Failure to recover renal function has negative effects on quality of life and health care costs [34]. Identification of patients at high risk for failure to recover has important implications for their long-term management, e.g. avoidance/minimization of nephrotoxins, early referral to a nephrologist. Despite significant advances in the epidemiology of AKI, the prediction of renal recovery from AKI remains a major clinical challenge. Unfortunately, no reliable method to predict renal recovery exists. Recent studies discovered biomarkers predicting recovery from AKI, but the prediction performance was limited [35], [36]. In the present study, we evaluated whether the course of [TIMP-2]*[IGFBP7] concentration can predict renal recovery. We defined renal recovery from AKI as a serum creatinine level at hospital discharge equal to or lower than the baseline creatinine level. In our study, the course of [TIMP-2]*[IGFBP7] concentrations proved a high sensitivity and specificity for the prediction of renal recovery from AKI after cardiac surgery. In contrast to [TIMP-2]*[IGFBP7], urinary NGAL concentration failed to predict renal recovery after cardiac surgery (Figure 4). Our data are in line with another study that demonstrated that NGAL has only limited ability to predict renal recovery [35]. However, a recently published study showed that in critically ill patients a panel of urine biomarkers can augment prediction of recovery after AKI [36]. This study evaluated six markers representing three aspects of the physiology of renal recovery: inflammatory, renal tubular epithelial cell regeneration, and filtration and tubular reabsorption markers.

Several molecules are known to be involved in the pathogenesis of AKI [37], [38]. IGFBP7 and TIMP-2 are both inducers of G_1_ cell cycle arrest, a mechanism involved in the early phase of AKI [20], [22]. After stress, injury or cell damage, renal tubular cells enter for a short period G_1_ cell-cycle arrest [19] until the damage has been repaired [21]. Cell cycle arrest occurs early after a variety of insults [22]. This may help explain the early increase of TIMP-2 and IGFBP7 in the urine of patients with AKI after CPB. The fact that the rapid decrease of [TIMP-2]*[IGFBP7] predicts recovery from AKI after cardiac surgery suggests that the duration of the stress and injury to the kidney after cardiac surgery is an important determinant of AKI severity and recovery. Consequently, early detection and risk assessment could improve patient outcome by means of early intervention and optimization of patient management [27].

Urinary biomarkers are frequently normalized against urinary creatinine to account for changes in urine flow rate. However, a timed collection of urine samples appears equally important for an accurate estimation of the excretion rate [39]. In our study, the first indication of AKI was based on oliguria in two thirds of patients. Normalization of [TIMP-2]*[IGFBP7] to urine creatinine concentration, however, did not change informative value.

Our study has several strengths. First, our cohort of patients was heterogeneous with significant comorbidities related to AKI, including patients with CKD. Although different biomarkers have a restricted accuracy in predicting the development of AKI in such a patient population [31], TIMP-2 and IGFB7 showed a very good performance in predicting AKI. Second, in contrast to other known biomarkers, [TIMP-2]*[IGFB7] has a very high sensitivity and specificity not only in diagnosing AKI, but also in predicting renal recovery in cardiac surgery patients. These results imply that co-interventions (e.g. application of radiocontrast agents) in patients who have a high risk of non-recovery of renal function can be adjusted or rejected. Preventing a second insult in high risk patients could hamper an aggravation of renal function or improve renal recovery and subsequently improve long-term outcome (renal function and survival).

There are several limitations to our study. First, our study represents the results from a relatively small number of patients from a single center. It is acknowledged that our results, although of clear clinical and statistical significance, will need to be validated in a larger population. This is important, because previous biomarker studies [40] that started in small cohorts showed extraordinary results were outliners when subsequently studied in lager cohorts. Second, we are unable to provide long term data from these patients. It would have been very interesting to investigate renal function and mortality 90 days and 1 year after cardiac surgery. Third, renal recovery is not necessarily generalizable, because we tested the course of TIMP-2 and IGFBP7 concentrations early after a defined insult. In clinical practice, it is very uncommon to know the onset of AKI. Therefore, future studies have to investigate whether the course of TIMP-2 and IGFBP7 concentrations at later time points or after the onset of AKI can predict renal recovery.

In summary, our results indicate that TIMP-2 and IGFBP7 are early predictive urinary biomarkers of AKI after cardiac surgery. The combination of these two biomarkers may allow for the reliable early prediction of AKI at all times after CPB. The course of urinary TIMP-2 and IGFBP7 concentrations appears to be useful for predicting renal recovery following cardiac surgery.
